# Loganin substantially ameliorates molecular deficits, pathologies and cognitive impairment in a mouse model of Alzheimer’s disease

**DOI:** 10.18632/aging.203646

**Published:** 2021-10-23

**Authors:** Lulin Nie, Kaiwu He, Fengzhu Xie, Shifeng Xiao, Shupeng Li, Jia Xu, Kaiqin Zhang, Chen Yang, Li Zhou, Jianjun Liu, Liangyu Zou, Xifei Yang

**Affiliations:** 1 Shenzhen Key Laboratory of Modern Toxicology, Shenzhen Medical Key Discipline of Health Toxicology (2020–2024), Shenzhen Center for Disease Control and Prevention, Shenzhen 518055, China; 2 School of Chemical Biology and Biotechnology, Peking University Shenzhen Graduate School, Shenzhen 518055, China; 3 Shenzhen Center for Chronic Disease Control and Prevention, Shenzhen 518020, China; 4 Shenzhen Key Laboratory of Marine Biotechnology and Ecology, College of Life Sciences and Oceanography, Shenzhen University, Shenzhen 518060, China; 5 Department of Pathophysiology, Guangzhou Medical University, Guangzhou 510182, China; 6 College of Public Health, University of South China, Hengyang 421001, China; 7 Department of Neurology, Shenzhen People’s Hospital, Second Clinical College, Jinan University, Shenzhen 518020, China

**Keywords:** Alzheimer's disease, 2D-DIGE, loganin, cognitive impairment

## Abstract

Alzheimer’s disease (AD) is the most common age-related neurodegenerative disease threatening the health of the elderly, but the available therapeutic and preventive drugs remain suboptimal. Loganin, an iridoid glycoside extracted from *Cornus officinalis*, is reported to have anti-inflammatory and memory-enhancing properties. This study is aimed to explore the influence of loganin on cognitive function in 3xTg-AD mice and the underlying mechanism associated with its neuroprotection. According to the results of behavioral tests, we found that administration of loganin could significantly alleviate anxiety behavior and improve memory deficits of 3xTg-AD mice. Furthermore, immunohistochemical analysis displayed that there were decreased Aβ deposition in the hippocampus and cortex of 3xTg-AD mice treated with loganin compared with the control mice. Importantly, the Aβ-related pathological change was mainly involved in altering APP expression and processing. And loganin was also found to reduce the levels of phosphorylated tau (i.e. pTau^S396^ and pTau^S262^) in 3xTg-AD mice. By performing 2D-DIGE combined with MALDI-TOF-MS/MS, we revealed 28 differentially expressed proteins in the 3xTg-AD mice treated with loganin compared with the control mice. Notably, 10 proteins largely involved in energy metabolism, synaptic proteins, inflammatory response, and ATP binding were simultaneously detected in 3xTg-AD mice compared to WT mice. The abnormal changes of energy metabolism (PAGM1 and ENO1), synaptic proteins (SYN2 and Cplx2), inflammatory response (1433Z) were verified by western blot. Overall, our study suggested that loganin could be used as a feasible candidate drug to ameliorate molecular deficits, pathologies and cognitive impairment for prevention and treatment of AD.

## INTRODUCTION

Alzheimer’s disease (AD) is a common form of dementia that nowadays influences more than 50 million people around the world and the majority of them are elderly [1]. It is accompanied with severe memory loss and characterized by obviously neuropathological changes including Aβ plaque depositing, Tau phosphorylation, synapse impairment, neuroinflammation [2–4]. Based on the World Alzheimer Report 2018 [1], there will be a new dementia patient every 3 seconds around the world, and this number will more than triple to 152 million by 2050. Moreover, the total global cost of dementia is estimated at 1 trillion dollars in 2018 and will rise to 2 trillion dollars by 2030. What is more serious is that there are virtually no effective treatments, so some researches focusing on the testing of active drugs for prevention and treatment of AD are necessarily carried out.

Loganin is a major iridoid glycoside extracted from *Cornus officinalis* that is a traditional Chinese herb. Studies *in vitro* and *in vivo* have shown that loganin is basically nontoxic or low toxicity [5, 6]. Reversely, numerous beneficial characteristics of loganin have been reported including anti-inflammatory and anti-tumor properties, as well as memory-enhancing activity. For example, loganin exerted significantly anti-inflammatory effect in MPTP-induced PD mice [7]. The mechanism of loganin-exhibited anti-inflammatory effect was partially realized through down-regulated NF-κB signaling pathway that reduced the release of proinflammatory cytokines such as IL-1β, IL-6, and TNF-α [8, 9]. Specifically, loganin had been demonstrated to protect PC12 cells from Aβ-caused inflammatory response *in vitro* [10]. In addition, loganin treatment could increase long-term potentiation (LTP) in cultured brain section, suggesting its involvement in improving memory [11]. In line with this work, a previous paper showed that loganin was administrated in a dose-dependent manner to improve learning and memory impairment caused by scopolamine [12]. However, the studies available on how loganin mechanistically affects learning and memory are still very rare.

The two-dimensional fluorescence differential gel electrophoresis (2D-DIGE) method is a valuable approach for proteomics [13]. Compared with traditional 2D approaches, the main advantage of the technique is that samples can be pooled. Besides, it can normalize for experimental variations in spot intensities and gel patterns based on special internal standard. 2D-DIGE technique is widely used in mechanism research. In present study, we assessed the effects of loganin on animal behavior including anxiety, memory, and neuropathology in the 3xTg-AD mice. Furthermore, to investigate the possible mechanisms of loganin-induced neuroprotective effect, we explored the influence of loganin treatment on hippocampal protein expression profile via 2D-DIGE combined with Matrix-Assisted Laser Desorption / Ionization Time of Flight Mass Spectrometry (MALDI-TOF-MS/MS).

## RESULTS

### Loganin alleviated anxiety behavior and prevented cognitive impairment of 3xTg-AD mice

**Figure 1:**
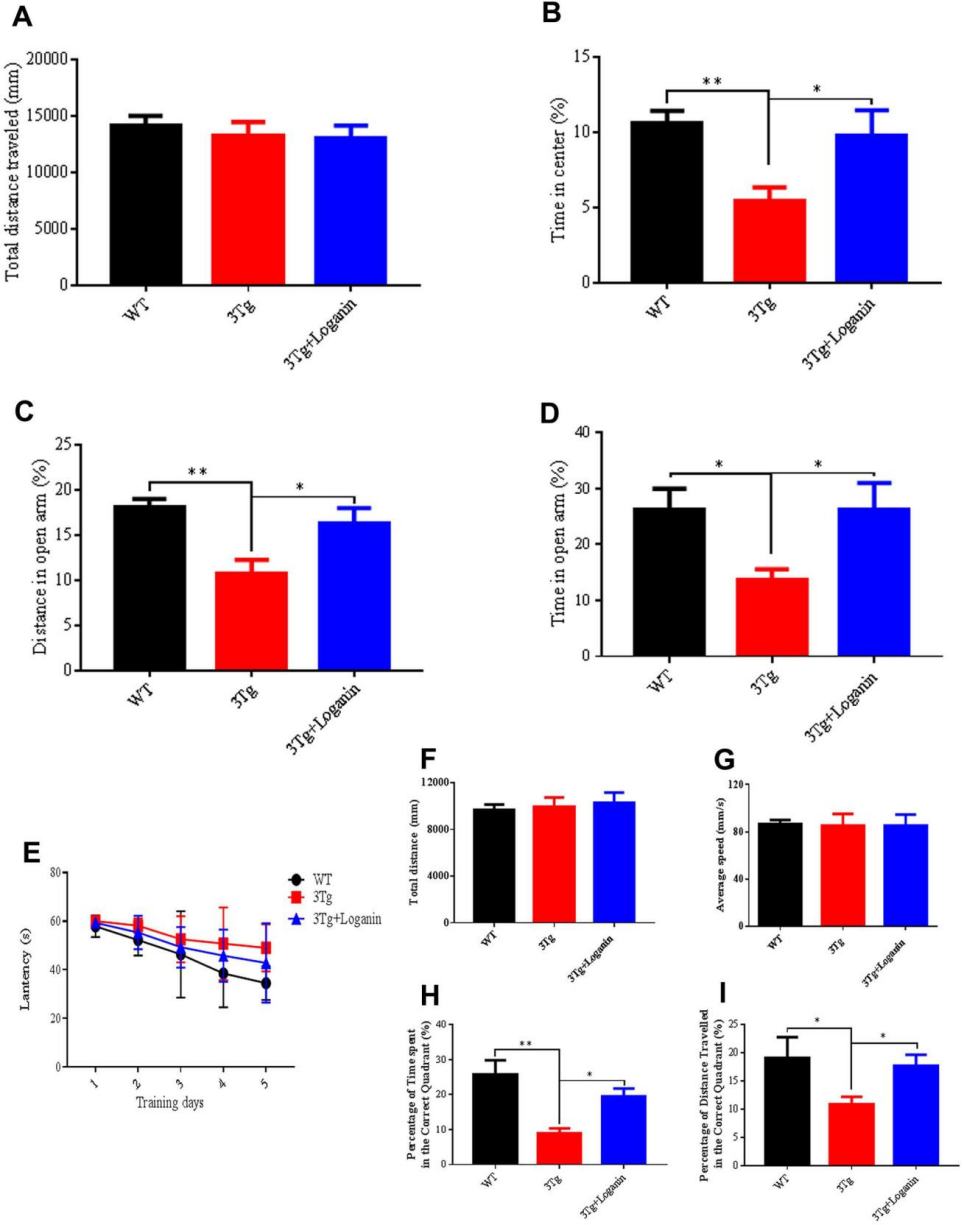
**Effects of loganin on anxiety and memory behavior.** (**A**) Total distance traveled in the open field. (**B**) The percentage of time spent in the center of the open field. (**C**) The percentage of distanced traveled in the open arm of the elevated plus maze. (**D**) The percentage of time spent in the open arm of the elevated plus maze. (**E**) The escape latency. (**F**) Total distance traveled in probe trial of Morris water maze. (**G**) Average speed in the probe trial of Morris water maze. (**H**) The percentage of time spent in the target quadrant. (**I**) The percentage of distance traveled in the target quadrant. Data were expressed as mean ± SEM, ^*^p < 0.05, ^**^p < 0.01, n = 10 - 15 for each group.

To assess if administration of loganin could influence psychiatric symptoms of 3xTg-AD mice, the open field test (OPT) and elevated plus maze (EPM) were performed to investigate the anxiety level in 3xTg-AD mice. The total distance travelled was no significance in the three groups (Figure 1A), indicating that the locomotivity of mice was good. Compared to WT mice, the time in center (%) of 3xTg-AD mice was significantly decreased, and the 3xTg-AD mice treated with loganin compared with the control mice spent significantly longer time in the central area of the open field (Figure 1B). Consistent with the results in the OPT, the data of the EPM showed that the distance and time in open arm (%) were significantly decreased in 3xTg-AD mice relative with WT mice (Figure 1C, 1D). After loganin treatment, the 3xTg-AD mice displayed significantly increased the distance and time in open arm (%). These data indicated that the 3xTg-AD mice displayed obvious anxiety behavior, while administration of loganin could significantly decreased anxiety level of 3xTg-AD mice.

To assess the influence of administration of loganin on cognitive ability of 3xTg-AD mice, we measured the spatial learning and memory abilities of the mice by preforming Morris water maze (MWM). In training trial, the 3xTg-AD mice spent obviously more time to search for the hidden platform compared to WT mice. In contrast, loganin treatment decreased the time spent in exploring hidden platform relative with the control mice (Figure 1E). In probe trial, the total distance and average speed have no significant differences in the three groups (Figure 1F, 1G). While the time spent and the distance traveled in the targeted quadrant were significantly decreased in the 3xTg-AD mice relative with WT mice (Figure 1H, 1I). Conversely, loganin-treated 3xTg-AD mice displayed much better performance in target recognition when compared with the control mice (Figure 1H, 1I). These results suggested that administration of loganin improved spatial learning and memory and prevented cognitive impairment in 3xTg-AD mice.

### Loganin attenuated Aβ deposition, improved tau pathology, and altered APP expression and processing in 3xTg-AD mice

**Figure 2:**
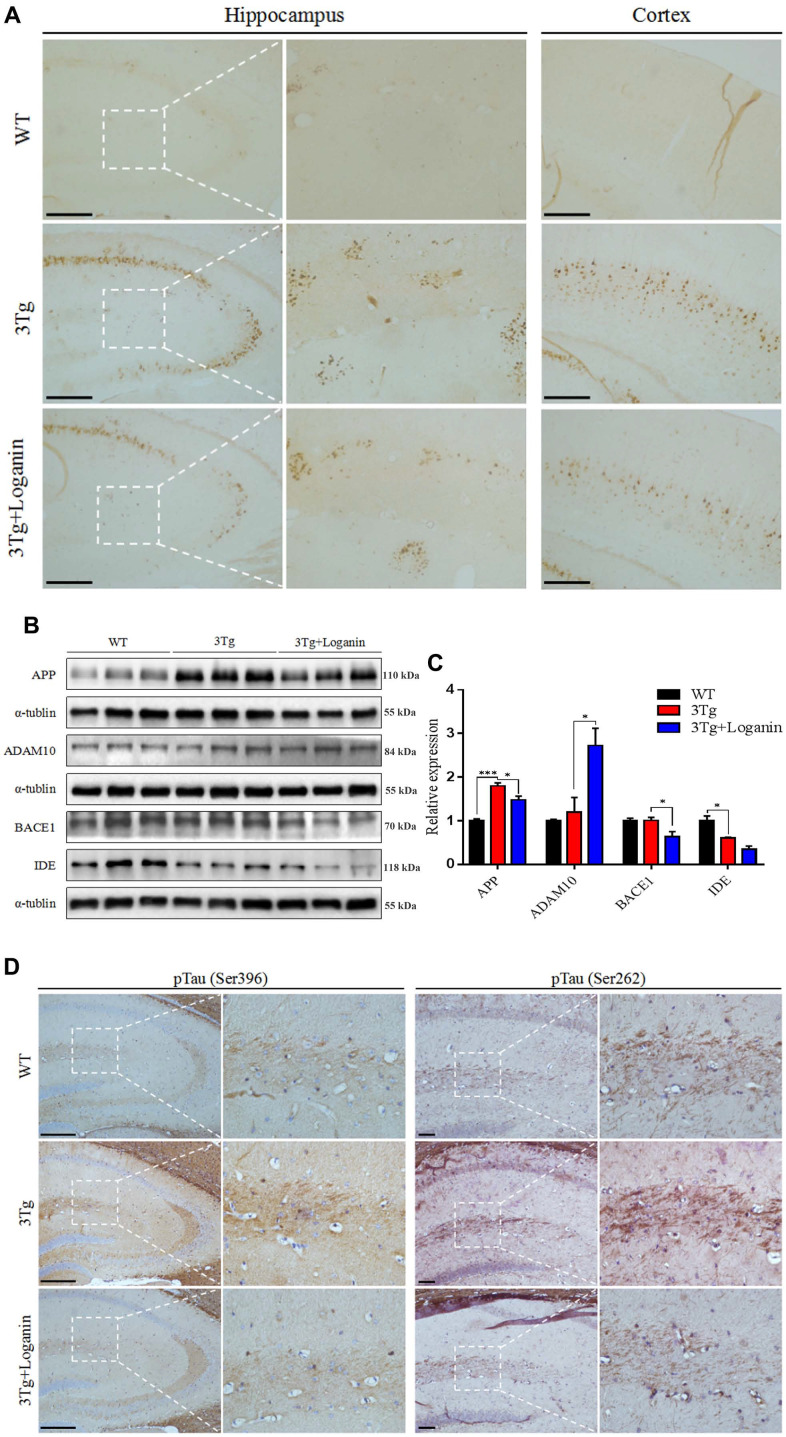
**Effects of loganin on AD pathology and the expression of Aβ-related key proteins.** (**A**) The relative expression of Aβ deposition. (**B**, **C**) The relative expression of APP expression and processing related proteins. (**D**) The relative expression of Tau phosphorylation. Scale bar = 100 μm. Data were expressed as mean ± SEM, ^*^p < 0.05, ^***^p < 0.001, n = 3 for each group.

To investigate if loganin treatment could produce beneficial effects on alleviation of Aβ pathology, one of the most important hallmarks of AD. To measure Aβ deposition in 3xTg-AD mic, brain sections from the three group mice were stained with 6E10 antibody. Compare with WT mice, Aβ deposition was significantly increased in the 3xTg-AD mice. While the 3xTg-AD mice treated with loganin displayed obviously decreased of Aβ deposition in the hippocampus and the cerebral cortex (Figure 2A). In order to further analyze how Aβ deposition was influenced by loganin, we detected a series of key proteins involved in the regulation of Aβ production, including APP, ADAM10, BACE, and IDE. Compared to the control mice, we found that there was no significant alteration in the expression level of IDE in the loganin-treated 3xTg-AD mice, but the expression levels of APP, ADAM10 and BACE1 were remarkable change in the 3xTg-AD mice treated with loganin (Figure 2B, 2C). Together, these findings suggested that loganin treatment attenuated Aβ deposition with altering APP expression and processing in 3xTg-AD mice.

To determine whether Tau protein phosphorylation was decreased in loganin-treated 3xTg-AD mice, we evaluated the levels of pTau^S396^ and pTau^S262^ in the hippocampal brain sections by immunohistochemistry. The data displayed that the 3xTg-AD mice had increased Tau phosphorylation at both serine^396^ and serine^262^ in hippocampus relative with WT mice. However, loganin treatment displayed a decreasing trend of the levels of Tau phosphorylation in 3xTg-AD mice treated with loganin (Figure 2D).

### Differentially expressed hippocampal proteins of 3xTg-AD mice with or without loganin treatment

**Figure 3:**
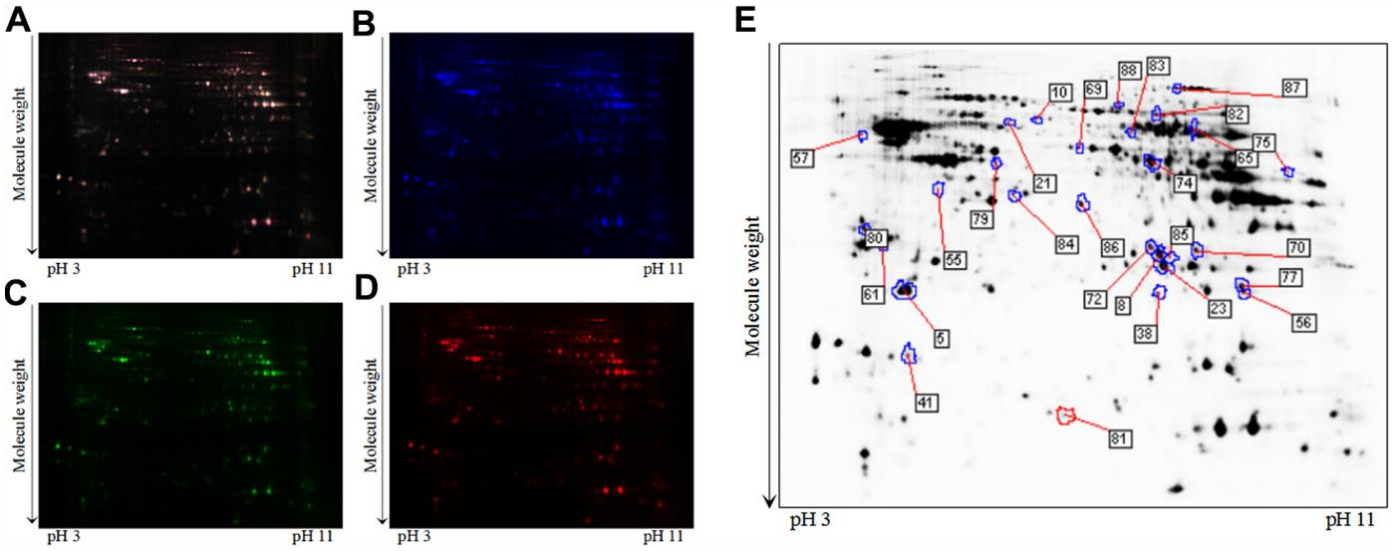
**A typical 2D-DIGE gel images of hippocampal proteins obtained from the 3xTg-AD mice treated with or without loganin (n = 6 for each group).** (**A**) The merged image displaying Cy2-, Cy3-, and Cy5-labeled proteins. (**B**) the typical gel image of Cy2-labeled proteins. (**C**) the typical gel image of Cy3-labeled proteins of the 3xTg-AD mice. (**D**) the typical gel image of Cy5-labeled proteins of the 3xTg-AD mice treated with loganin. (**E**) Greyscale 2D-DIGE gel image showing the differentially expressed proteins from the hippocampus of 3xTg-AD mice treated with loganin compared to the control mice.

To further explore how loganin displayed anti-AD activity in 3xTg-AD mice, 2D-DIGE technique coupled with MALDI-TOF-MS/MS was employed to perform proteomics. Representative 2D-DIGE gel images of hippocampal samples isolated from the 3xTg-AD mice with or without loganin treatment were showed in Figure 3A–3D. Protein spots with a p-value ≤ 0.05 were defined as differentially expressed protein spots. A total of 28 differentially expressed protein spots were selected (Figure 3E) and identified by the method of MALDI-TOF-MS/MS. Finally, combined with the results of searching database, these differentially expressed proteins were showed in Supplementary Table 1.

### Functional categories and heatmap analysis of the differentially expressed proteins

**Figure 4:**
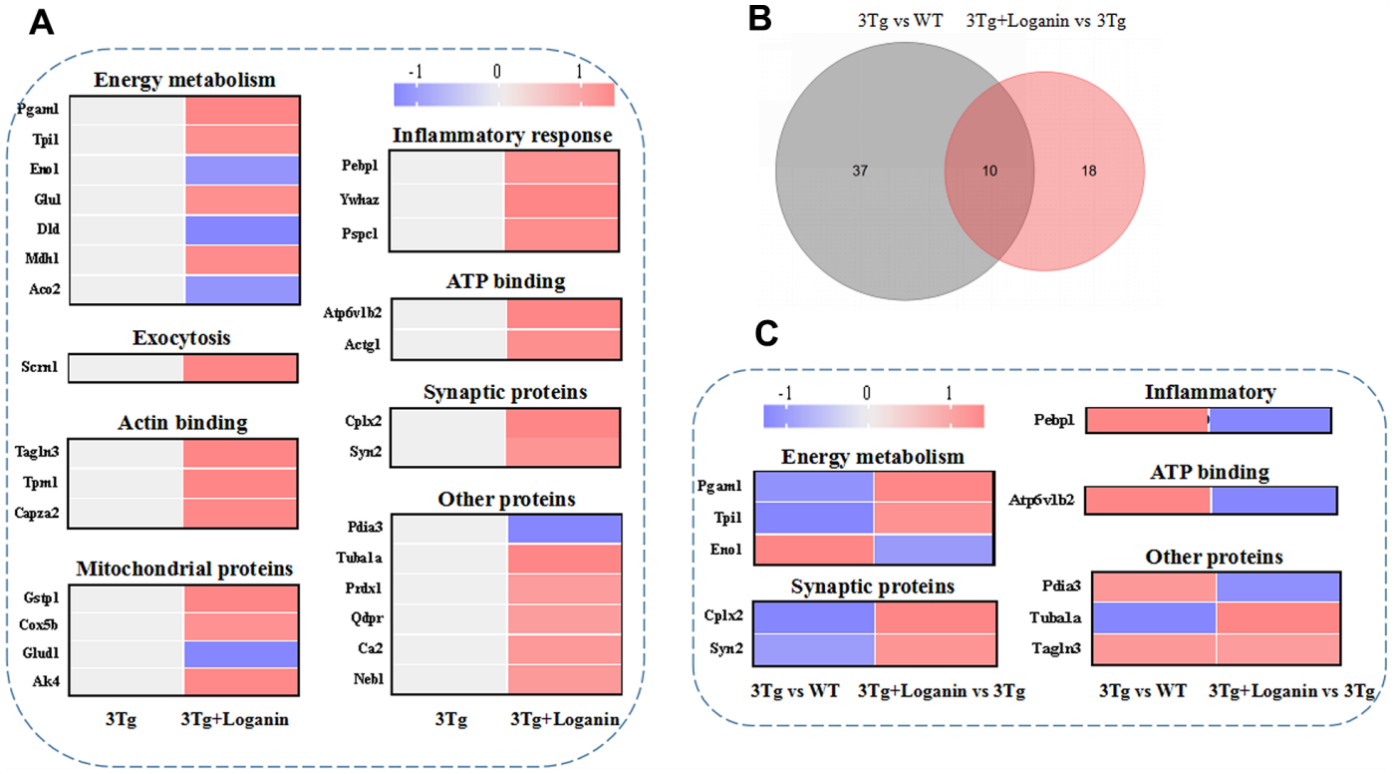
**Functional categories, heatmap analysis and Venn diagram analysis.** (**A**) Functional categories and heatmap analysis of 28 differentially expressed proteins from the 3xTg-AD mice treated with loganin compared to the control mice. (**B**) The Venn diagram analysis. (**C**) Functional categories and heatmap analysis of the co-differentially expressed proteins among three groups. Red expressed up-regulated and blue expressed down-regulated. The brighter the image, the greater the fold change.

To determine the functional categories of differentially expressed proteins from the 3xTg-AD mice treated with loganin compared with the control mice, a web-based portal called metascape that was an effective and efficient tool for omics analysis was used to analyze the function of proteins. Combined with heatmap analysis, these differentially expressed proteins from the loganin-treated 3xTg-AD mice compared with the control mice were mainly divided into following categories, including energy metabolism, exocytosis, actin binding, mitochondrial proteins, inflammatory response, ATP binding, synaptic proteins, and other proteins (Figure 4A). And red expressed upregulation and blue expressed downregulation, and the greater the fold change, the brighter the image. In addition, 10 differentially expressed proteins found in the three groups were further divided into the following function including energy metabolism, synaptic proteins, inflammatory response, ATP binding and other proteins (Figure 4B, 4C). These data indicated that some functional clustering (i.e. energy metabolism, synaptic proteins, and inflammatory response) may be play an important role in loganin-caused neuroprotection of 3xTg-AD mice.

### GO annotation enrichment analysis of the differentially expressed proteins

**Figure 5:**
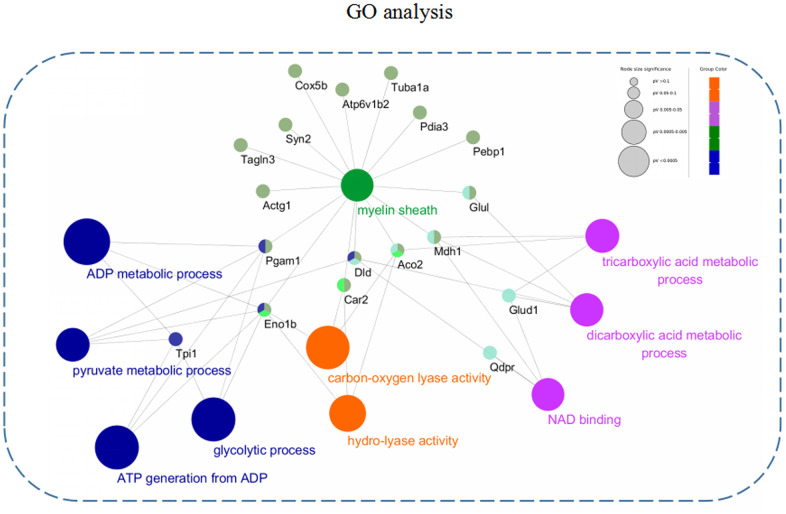
**GO annotation enrichment analysis.** The differentially expressed proteins from the 3xTg-AD mice treated with loganin compared with the control mice were enriched in BP, MF, and CC through a Cytoscape plug-in ClueGo. Different colour represented main classification of GO enrichment, and the bigger the circle changed, the better the enrichment.

In order to further describe the distributions of GO annotation enrichment of the differentially expressed proteins. A Cytoscape plug-in ClueGo (version 2.5.1) was used to conduct and visualize functionally grouped network of terms involving in biological process (BP), molecular function (MF), and cellular component (CC). As shown in Figure 5, these differentially expressed proteins from the 3xTg-AD mice treated with loganin compared with the control mice were enriched in following subterms including ADP metabolic process, pyruvate metabolic process, glycolytic process, ATP generation from ADP, carbon-oxygen lyase activity, hydro-lyase activity, tricarboxylic acid metabolic process, dicarboxylic acid metabolic process, NAD binding, and myelin sheath. Different colour represented main classification of GO enrichment, and the bigger the circle changed, the better the enrichment. Notably, the different subterms were closely linked by some differentially expressed proteins such as Eno1, Pagm1, Car2, Dld, Aco2, Mdh1, and Glu1, suggesting that loganin-induced neuroprotective effects may be regulated by these molecules above.

### STRING analysis of the differentially expressed proteins

**Figure 6:**
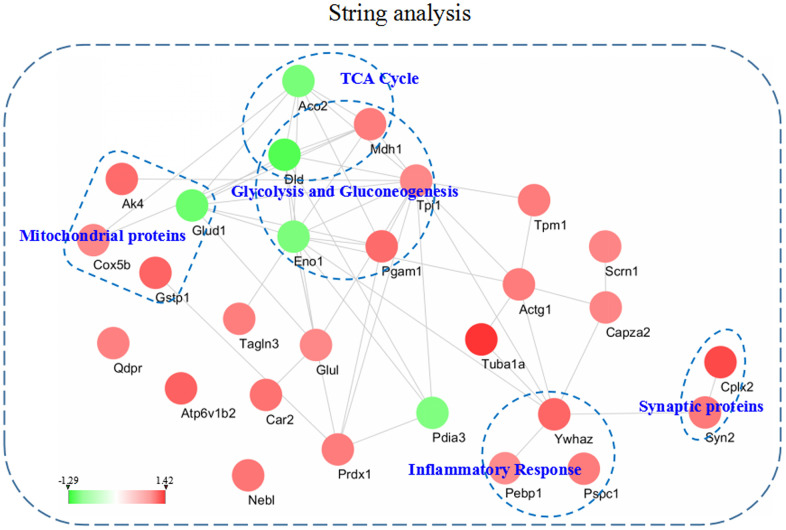
**The intricate PPI network of the differentially expressed proteins and selected functional categories.** This network and functional categories were conducted and visualized among the dysregulated proteins in the hippocampus of the 3xTg-AD mice treated with loganin compared with the control mice. Red expressed up-regulated and green expressed down-regulated. The brighter the image, the greater the fold change.

To better understand the functional connectivity of the differentially expressed proteins, we used the STRING linked with Cytoscape 3.6.1 to plot and visualize the PPI network. Some molecules from the 3xTg-AD mice treated with loganin compared with the control mice involved in TCA cycle, glycolysis and gluconeogenesis, mitochondrial proteins, inflammatory response, and synaptic proteins were differentially affected and obviously highlighted in PPI network. As was shown in Figure 6, the PPI network again indicated that some differentially expressed proteins such as Enol, Pagm1, etc. were closely related with other molecules.

### Validation of the differentially expressed proteins by western blot

**Figure 7:**
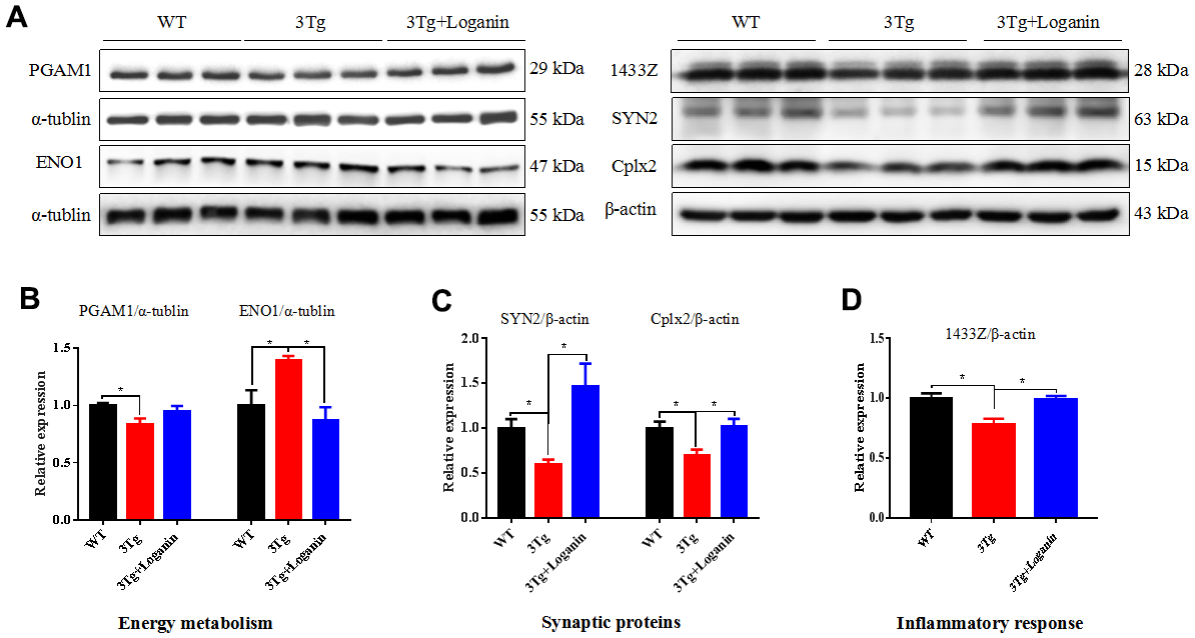
**Validation of key proteins involving in neuroprotective effects of loganin.** (**A**–**D**) The relative expression of energy metabolism, synaptic proteins, and inflammatory response related proteins in WT mice and 3xTg-AD mice with or without loganin treatment. Data were expressed as mean ± SEM, ^*^p < 0.05, n =3 for each group.

According the results of proteomics analysis, we found that energy metabolism, synaptic proteins, and inflammatory response were abnormally regulated by loganin treatment in the 3xTg-AD mice. Furthermore, the expression levels of several key proteins (i.e. PAGM1, ENO1, SYN2, Cplx2, and 1433Z) involving in the above three aspects were validated by western blot. Compared to the control mice, the expression of PAGM1, SYN2, Cplx2, and 1433Z were determined to be upregulated and the expression of ENO1 was determined to be downregulated in the 3xTg-AD mice treated with loganin (Figure 7A–7D). The data indicated that the results of western blot were consistent with that of proteomics.

## DISCUSSION

Clinical trials showed that cognitive impairment was usually accompanied by neuropsychiatric symptoms (i.e. anxiety and depression) in AD [14]. In this study, the 3xTg-AD mice, a classic AD model, were selected to comprehensively evaluated the influence of loganin on the behavior of mice, including neuropsychiatric symptoms (i.e. anxiety behavior) and cognitive impairment (i.e. learning and memory). Although it was reported that loganin possibly alleviated anxiety- and depression-like behaviors related with diabetes via reducing levels of blood glucose and pro-inflammatory cytokine [15]. And also, previous studies had been demonstrated that loganin could effectively decrease long-term potentiation caused the blockade of cholinergic muscarinic receptor and recover scopolamine-caused learning and memory deficits [11], and the results of diabetic male rats showed that acute administration of loganin could significantly alleviate diabetes-induced spatial memory impairment [16]. However, there were no reported in the classical AD models such as 3xTg-AD, APP/PS1, pR5, etc. Notably, our research filled this gap involving in loganin-induced neuroprotection in 3xTg-AD mice.

Aβ deposition and Tau phosphorylation is the two characteristic pathological changes of AD [2, 17]. In present study, we investigated the effect of loganin treatment on AD pathology in 3xTg-AD mice. Our data showed that loganin significantly alleviated AD pathology such as reducing Aβ deposition and decreasing the level of pTau^S396^ and pTau^S262^ in the brain of 3xTg-AD mice. Although the effect of loganin on AD pathology had not been reported in previous studies, an iridoid glucoside called catalpol that was structurally very similar to loganin was found to be able to decrease the levels of soluble Aβ40 and Aβ42 in cell experiment and thus suppress the accumulation of senile plaques [18]. In addition, geniposide, another structural analog of loganin, was reported to significantly decrease tau hyperphosphorylation in STZ-induced sporadic AD rats [19]. These data suggested that loganin may have also similar neuropharmacological effects in anti-AD pathology.

Our study was the first to explore the possible mechanism of the neuroprotective effects of loganin as a potential candidate drug for AD that could ameliorate Aβ deposition, improve tau pathology and prevent cognitive impairment in 3xTg-AD mice at the level of hippocampal proteomics. Though loganin had been found to exhibit significant neuroprotection and the property of sluggishing the process of neurodegeneration in AD [20]. Besides, previous study was also reported that loganin showed strong inhibitory activity against β-secretase (BACE1) *in vitro* assay [21], and the result of BACE1 inhibitory property was consistent with our data *in vivo*. However, there was few studies focusing on the mechanism of the neuroprotective effects of loganin. In this study, the results of proteomics analysis indicated that the differentially expressed proteins involving in energy metabolism, synaptic proteins, and inflammatory response may directly or indirectly regulate cognitive impairment in 3xTg-AD mice. Therefore, we would discuss the possible mechanisms of the neuroprotective effects of loganin for AD in the following aspects.

### Energy metabolism

In AD, the major manifestations were closely linked with impairment of energy metabolism that mainly included glycolysis and tricarboxylic acid cyclic (TCA) [22, 23]. Moreover, more and more newly developed drugs for AD highlighted the improtant role of perturbations in cellular energy metabolism in the pathophysiology [24]. In this study, proteomics analysis revealed that several proteins involving in energy metabolism were differentially expressed in hippocampus of 3xTg-AD mice treated with loganin compared with the control mice. Phosphoglycerate mutase 1 (PGAM1), an glycolytic enzyme, was involved in glycolysis, pentose phosphate pathway, and serine synthesis via regulating the conversion of 3-phosphoglycerate (3PG) to 2-phosphoglycerate (2PG) [25]. Alpha-enolase (ENO1) that catalyzed the conversion of 2-phosphoglycerate (2PG) to phosphoenolpyruvate (PEP) was one of only two glycolytic enzymes consistently up-regulated from MCI to AD [26]. And it was also recommended as a potential therapeutic target for AD [27]. Overall, loganin treatment for 3xTg-AD mice caused the abnormal expression of glycolysis-related proteins such as PAGM1 and ENO1, which could accelerate the production of pyruvate and thus indirectly increase the supply of ATP.

### Synaptic proteins

The synaptic dysfunction and synapse loss that were largely regulated by the expression of synaptic proteins contributed to the cognitive impairment in patients with AD [28]. In this study, our data revealed that the expression of two synaptic proteins (i.e. synapsin-2 (SYN2) and complexin-2 (Cplx2)) were protectively up-regulated in hippocampus of 3xTg-AD mice treated with loganin compared with the control mice. It had been reported that the lowering expression of SYN2 induced by oligomeric α-synuclein exacerbated cognitive impairment in an AD mouse model [29]. Besides, the reduction of SYN2 was also thought to disrupt the release of neurotransmitters, which caused synaptic dysfunction and cognitive deficits [30]. In addition, it was reported that Cplx2 was involved in the regulation of cognitive abilities in old age through the soluble N-ethylmaleimide-sensitive factor attachment protein receptor (SNARE) interactome [31], and it also promoted the transport of vesicles to the presynaptic membrane by regulating the neurotransmitter release [32]. Taken together, our finding indicated that loganin prevented cognitive impairment related with the increased expression of SYN2 and Cplx2 that were involved in the regulation of synaptic dysfunction.

### Inflammatory response

In the center nervous system, the inflammatory response was usually accompanied by activation of glial cells such as microglia and astrocytes that played an important role in neurodegenerative diseases [33]. 14-3-3 protein mainly including seven isoforms was abundantly expressed in different glial cells [34], and its expression was down-regulated in AD [35]. Interestingly, our study showed that the expression of 14-3-3Z, an inflammatory signaling protein, was significantly increased in the 3xTg-AD mice treated with loganin compared with the control mice. Combined with previous studies, we speculated that the up-regulation of 14-3-3Z may inhibit the inflammatory response mediated by glial cells and thus induced neuroprotective effects in 3xTg-AD mice. Of course, more evidence would be needed to provide to support our guess.

## CONCLUSIONS

**Figure 8:**
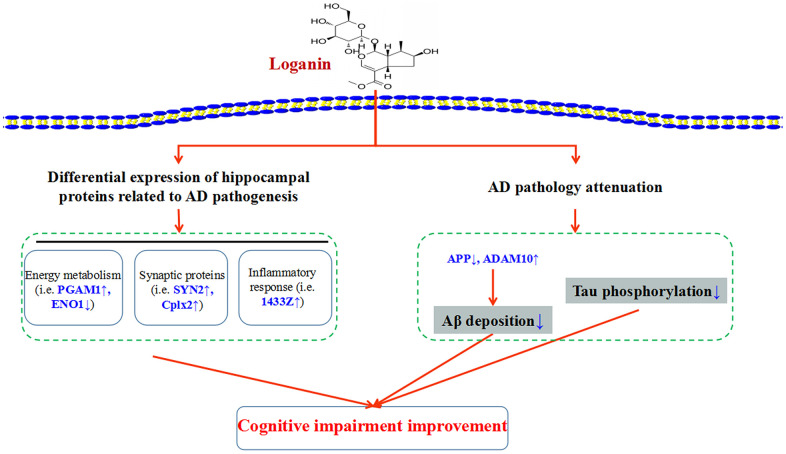
**Schematic diagram demonstrating the proposed potential mechanisms involving in neuroprotective effects of loganin.** Administration of loganin induced the change of hippocampal proteome and AD pathology. Obviously, the abnormal expression of the differentially expressed proteins may directly or indirectly regulate cognitive impairment of the 3xTg-AD mice.

As depicted in Figure 8, our data suggested that loganin could ameliorate Aβ deposition, improve tau pathology and prevent cognitive impairment in 3xTg-AD mice. Proteomics analysis further elucidated the possible mechanism of the loganin-induced neuroprotective effects of 3xTg mice. Namely, these differentially expressed proteins from the 3xTg-AD mice treated with loganin compared to the control mice may directly regulate cognitive deficits of the 3xTg-AD mice via modulating the expression of key molecules (i.e. PAGM1, ENO1, SYN2, Cplx2, and 1433Z), and may be indirectly involved in the regulation of AD pathology and thus affected cognitive impairment of the 3xTg-AD mice. Therefore, our study provided more evidence that loganin could be used as a feasible candidate drug for the prevention and treatment of AD.

## MATERIALS AND METHODS

### Regents and antibodies

Loganin was obtained from Prof. Zhendan He, Shenzhen Univeristy. Immunohistochemistry kit (ab64264), anti-APP (ab32136), anti-ADAM10 (ab39178), anti-IDE (ab32216), anti-pTau^S396^ (ab109390), anti-Cplx2 (ab232895), anti-PAGM1 (ab184232), anti-ENO1 (ab155102), anti-SYN2 (ab76494), and anti-1433Z (ab51129) antibodies were purchased from Abcam. Anti-β-actin (sc-47778) antibody was purchased from Santa Cruz Biotechnology. Anti-BACE1 (5606S) was purchased from Cell Signaling Technology. Anti-β-Amyloid (Aβ) (6E10) (803015) antibody was purchased from BioLegend. Anti-pTau^S262^ (44-750G), Pierce^TM^ BCA protein assay kit (23225), Pierce^TM^ fast western blot kit, ECL substrate (35050), Goat anti-Mouse IgG (31430) and anti-Rabbit IgG (31460) secondary antibodies were purchased from Thermo Fisher Scientific. Anti-α-tubulin (T9026) antibody, lysine (L5501), urea (U5378), thiourea (T7875), CHAPS (C9426), DL-dithiothreitol (DTT) (43815), 3-indoleacrylic acid (IAA) (I1625), sodium dodecyl sulfate (SDS) (436143), phosphoric acid (438081), ammonia sulfate (901874), trypsin (T2600000), acetonitrile (ACN) (34967), trifluoroacetic acid (TFA) (3020331), and α-Cyano-4-hydroxycinnamic acid (CHCA) (476870) were purchased from Sigma-Aldrich. 2-D Quant kit (80-6483-56), CyDye^TM^ DIGE Fluor minimal labeling kit (25-8010-65), IPG buffer, pH 3~11 NL (17-6004-40), Immobiline DryStrip pH 3~11 NL, 24 cm (17-6003-77), and glycerol (17-1325-01) were purchased from GE Healthcare. Bromophenol blue (161-0404) and Coomassie brilliant blue G-250 (1610406) were purchased from Bio-Rad. The usages of primary antibodies were listed in Supplementary Table 2.

### Animals and treatment

The 3xTg-AD mice harboring three mutant alleles (APP_Swe_, PS1_M146V_, and Tau_P301L_) were purchased from Jackson Laboratory and kept in animals room of Shenzhen Center for Disease Control and Prevention (SZCDC). All animals were maintained specific conditions (temperature: 22 ± 2° C, relative humidity: 55 ± 15%) with free access to feed and water. In our experiment, the 7-month-old female mice were divided into three groups, and each group contained 10 to 15 mice. The 3xTg-AD mice were administrated daily with loganin (20 mg/kg body weight) for duration of 6 weeks. To avoid possible deviation to mice from long-term intraperitoneal injection, the control mice were injected with saline solution. Animal’s body weight was monitored every 3 days. After loganin treatment, mice were given to behavioral tests and then sacrificed for biochemical analysis. All animal care and experimental procedures were approved by the Ethics Committee of SZCDC. Every effort was made to alleviate animal’s suffering and minimize the number of mice used.

### Behavioral test

All tests were conducted in the behavioral room, beginning at 8:00 am every day. The mice were transferred to the specific room 2 h before the test. In present study, anxiety-related behaviors including open field test (OPT) and elevated plus maze (EPM) were performed to assess the level of animal’s anxiety, and memory-related behavior (i.e. Morris water maze (MWM)) was performed to evaluated cognitive ability of mice. A 3-day window was arranged between different behavioral experiments in order to avoid interest effects. The methods of testing were used as previously published studies [36].

### OPT

The open field arenas were 50 x 50 cm arenas with black Plexiglas walls. The whole bottom area of the apparatus was divided into 16 identical squares. The center of the arenas was defined as the inner 25 x 25 cm area and the corners of the arenas were defined as the 12.5 x 12.5 cm squares in each corner of the arena. Mice were placed in the middle of the open field, then allowed to explore the device for 5 min without interference from the outside environment. Xeye software was applied to record the total distance traveled and the time spent of the animals in the center, which could reflect the degree of anxiety. After each test, the device was fully cleaned with 75% ethanol.

### EPM

The EPM consisted of a raised platform with a central area (10 x 10 cm), two open arms (50 x 10 cm) and two closed arms (50 x 10 cm). The experimental mouse was placed in the center facing an open arm and allowed to explore freely the device for 5 min. Anxiety behavior was evaluated by measure the percentage of the time and distance of the experimental mice entering into open arms. Less time and distance in open arms represented the more anxiety of mice.

### MWM

MWM was applied to assess cognitive ability of the experiment mice. The maze mainly comprised a circular tank with a diameter of 1.7 m filled with no-fat milk (a depth of 0.3 m). The tank was divided into 4 quadrants (I - IV), and a circular white escape platform was placed 2 cm below the surface of the water in the third quadrant (target quadrant). Water temperature was maintained at 22 ± 1° C to prevent the mice from floating. In the acquisition training, all animals were trained one by one for five continuous days of testing with 4 trials per day. Each mouse was given 60 s to find the hidden platform in every trial, and the time to find the hidden platform was defined as the escape latency. If the mouse cannot find the platform within 60 s, and it was manually guided to stay on the hidden platform 15 s. After 1 week, the mouse was placed into the pool from the first quadrant after the hidden platform was remove. Each mouse was allowed to freely explore the pool for 2 min. All behavioral parameters of the experimental mice ware recorded using the video tracking system.

### Tissue processing and immunohistochemistry

After the behavioral experiments, all animals were deeply anesthetized with 4% chloral hydrate by intraperitoneal injection, and then perfused intracardially with saline solution. Their brains were immediately removed and split in two parts on the mid-sagittal plane. The hippocampus and cortex were separated from the left cerebral hemisphere, and stored at -80° C until use. The right cerebral hemisphere was transferred to fresh 4% paraformaldehyde overnight at 4° C. The fixed right hemibrains were dehydrated by gradient alcohol (75% ethanol, 2 h; 85% ethanol, 1 h; 95% ethanol, 1 h; 95% ethanol, 1 h; 100% ethanol, 0.5 h; 100% ethanol, 0.5 h; xylene, 0.5 h; xylene, 0.5 h; paraffin, 0.5 h; paraffin, 0.5 h; paraffin, 0.5 h), and then quickly embedded into paraffin blocks. 5 μm sections of these brain tissue were collected by using a paraffin microtome.

For subsequently immunohistochemical studies, the paraffin-embedded sections were carried out deparaffinized and rehydrated with xylene and ethanol. The levels of Aβ plaque and phosphorylated tau were measured according to the instruction of IHC kit. Briefly, these pretreated sections were added enough drops of hydrogen peroxide block to quenched endogenous peroxidase, and applied protein block to block nonspecific background staining. The slides were incubated at 4° C overnight with primary antibodies such as anti-6E10, anti-pTau^S396^, and anti-pTau^S262^. After washing with 0.1 M PBS in the next day, the sections were incubated with second antibody (i.e. biotinylated goat anti-polyvalent) for 1 h, followed by incubation with streptavidin peroxidase for 0.5 h. The sections were washed in 0.1 M PBS. Finally, the location of peroxidase was visualized by diaminobenzidine (DAB)-hydrogen peroxide substrate to give a brown color. The images were collected using a light microscope equipped with a digital camera (Olympus BX60, Japan).

### Proteomics analysis

### Sample preparation and protein labeling

Frozen hippocampal tissue was ultrasounded with DIGE-specific lysis buffer containing 7 M urea, 2 M thiourea, 4% CHAPS and 30 mM Tris-HCl, and then centrifuged at 4° C, 20000 g for 30 min. The supernatant was obtained and removed salts by ultrafiltration. The concentration of protein was assessed with 2-D Quant kit according to the instruction of manufacturer’s protocol.

6 hippocampal samples selected from each group were performed CyDyes labeling. An equal amount of total protein from all samples were mixed, and a total of 25 μg protein come from the mixture was regarded as an internal standard. According to the manufacture’s protocol, 200 pmol CyDye labeled 25 μg protein sample. Cy2 labeled the internal standard. In parallel, 25 μg protein from each experimental sample was labeled with either Cy3 or Cy5. After labeling on ice in the dark for 30 min, 10 mM lysine was added to stop the reaction. Then, the equal amount of Cy2-, Cy3- and Cy5-labeled protein samples were mixed together, and every mixture was added with an equal volume of 2 x lysis buffer containing 8 M urea, 2% CHAPS, 0.2% DTT, 2% (v/v) IPG buffer, pH 3-11 NL, 0.002% bromophenol blue. Finally, the total volume was replenished to 450 μL with rehydration buffer.

### 2D-DIGE and image analysis

In present study, 2D-DIGE was performed as previously described [36]. After overnight rehydration of the strips with 75 μg of mixed protein sample, the strips were carried out the first-dimension isoelectric focusing (IEF) by using the IEF system (GE Healthcare, USA). IEF was performed according to the following condition (i.e. 50 V, 18h; 300 V, 12 h; 500 V, 2 h; 1000 V, 2 h; 8000 V, 8 h). After IEF, strips were incubated with equilibration buffer (30% glycerol, 6 M urea, 75 mM Tris-HCl buffer (pH 8.8), 2% SDS) supplemented with 1% DTT for 15 min, followed with 4.5% IAA in same buffer for 15 min. The equilibrated strips were performed the second dimension SDS-PAGE with 12.5% SDS-PAGE gels. Then, 2D-DIGE gels were scanned on the Typhoon TRIO Variable Mode Imager (GE Healthcare, USA). To minimize the variation from different 2D-DIGE gels, PMT was adjusted to ensure that the maximum pixel intensity of each image was in the range of 40,000-60,000 pixels.

Based on the Differential In-gel Analysis (DIA) and the Biological Variance Analysis (BVA) modules, the DeCyder TM 2D software (version 6.5 GE Healthcare) was employed to analyzed these 2D-DIGE gels. The fluorescent intensity of each protein spot in the Cy3 or Cy5 was normalized with that of the corresponding Cy2 spot. The normalized fluorescent intensity of each spot in the gels was compared between the replicate groups. Finally, the differential protein spots with significant difference (*p* < 0 05) were marked to further analyzed.

### Protein identification

1200 μg of hippocampal protein was separated according to the identical method but without CyDyes labeling. Then the gels were incubated overnight with Coomassie brilliant blue solution (0.12% Coomassie brilliant blue G-250, 20% ethanol, 10% phosphoric acid, and 10% ammonia sulfate). Differentially expressed protein spots were manually picked from the staining gel. These pieces were subsequently destained and digested overnight in a trypsin buffer at 37° C, and then analyzed by MALDI-TOF-MS/MS.

For the MALDL-TOF-MS/MS, 0.6 μL peptide extract from each differential protein spot was added to a stainless steel target, and was subsequently treated with 1 μL 50% ACN and 0.1% TFA containing 10 mg/mL CHCA. After the spectra were manually calibrated, database searching was performed with the help of MASCOT based on the SwissProt databases in *Mus musculus*. Protein molecular weight and isoelectric point was recorded to assess the identification of protein spots.

### Bioinformatic analysis

The results of proteomic were analyzed by a variety of methods and approaches. Metascape (http://metascape.org/gp/index.html) was used to classify the functional categories of differentially expressed proteins, and Graphpad Prism 7.00 was employed to perform heatmap analysis. The Venn diagram plug-in in OriginPro 2019b software was applied to perform a logistic analysis among three groups. For the gene ontology (GO) annotation enrichment analysis, we used the ClueGO plug-in of the Cytoscape 3.6.1 software. STRING version 11.0 (https://string-db.org/) was employed to conduct the protein-protein interaction (PPI) network. Lastly, the Cytoscape 3.6.1 software was used to visualized these networks above.

### Western blot analysis

The hippocampal tissues from three groups were lysed with RIPA buffer, ultrasounded and centrifuged at 4° C, 14000 g for 30 min. The supernatants were obtained and quantified by using BCA protein assay kit. The degenerated protein lysates were isolated by 10% SDS-PAGE, transferred onto 0.22 μm PVDF membrane, blocked with 5% nonfat milk dissolved in 1 x TBST buffer, and incubated overnight with primary antibodies on ice and then with the corresponding secondary antibodies for 1 h at room temperature. Finally, protein chemiluminescence signal was measured by using ECL kit and quantified using Quantity One 4.6.2 software.

### Statistical analysis

All data was expressed as mean ± SEM and analyzed using GraphPad Prism 7.00 (Graphpad Software, Inc.). One-way analysis of variance (ANOVA) was employed to determine the statistical significance of differences among groups and following the Dunnett's multiple comparison test. A probability value of *p* < 0.05, *p* < 0.01, and *p* < 0.001 was considered statistically significant.

## Supplementary Material

Supplementary Tables
