# Supplementary material for: Loganin substantially ameliorates molecular deficits, pathologies and cognitive impairment in a mouse model of Alzheimer’s disease
Source: Aging (Albany NY). 2021 Oct 23;13(20):23739–56. doi: 10.18632/aging.203646 (PMC8580356; doi:10.18632/aging.203646)
Supplement: Supplementary Tables [file aging-13-203646-s001.pdf]

SUPPLEMENTARY TABLES

Supplementary Table 1. Differentially expressed hippocampus protein spots identified by 2D-DIGE coupled with MALDI-TOF-MS/MS from 3xTg-AD mice treated with loganin compared with the control mice.

Spot No[a].	Accession No.	Protein name[b]	MW (Da)[C]	Mascot score	3Tg+Loganin vs 3Tg	
					p-Value	Ratio[d]
5	PEBP1_MOUSE	Phosphatidylethanolamine-binding protein 1	20988	198	0.014	1.05
8	PGAM1_MOUSE	Phosphoglycerate mutase 1	28928	546	0.0015	1.19
10	PDIA3_MOUSE	Protein disulfide-isomerase A3	57099	241	0.017	-1.1
21	VATB2_MOUSE	V-type proton ATPase subunit B, brain isoform	56857	337	0.029	1.23
23	TPIS_MOUSE	Triosephosphate isomerase	32684	279	0.042	1.07
38	TAGL3_MOUSE	Transgelin-3	22627	155	0.037	1.11
41	CPLX2_MOUSE	Complexin-2	15499	112	0.024	1.33
55	TBA1A_MOUSE	Tubulin alpha-1A chain	50788	174	0.0083	1.42
56	B1AXW5_MOUSE	Peroxiredoxin-1 (Fragment)	19086	379	0.011	1.12
57	SCRN1_MOUSE	Secernin-1	46924	202	0.016	1.08
61	1433Z_MOUSE	14-3-3 protein zeta/delta	27925	151	0.0086	1.21
65	SYN2_MOUSE	Synapsin-2	63618	275	0.015	1.13
69	ENOA_MOUSE	Alpha-enolase	47453	136	0.03	-1.12
70	DHPR_MOUSE	Dihydropteridine reductase	25782	360	0.017	1.1
72	CAH2_MOUSE	Carbonic anhydrase 2	29129	195	0.0079	1.16
74	GLNA_MOUSE	Glutamine synthetase	42834	412	0.048	1.07
75	G5E8R0_MOUSE	Tropomyosin 1, alpha, isoform CRA_i	28383	275	0.032	1.12
77	GSTP1_MOUSE	Glutathione S-transferase P 1	23765	434	0.00028	1.22
79	ACTG_MOUSE	Actin, cytoplasmic 2	42108	176	0.021	1.12
80	LNEBL_MOUSE	LIM zinc-binding domain-containing Nebulette	31492	110	0.014	1.15
81	COX5B_MOUSE	Cytochrome c oxidase subunit 5B, mitochondrial	14089	222	0.016	1.07
82	DLDH_MOUSE	Dihydrolipoyl dehydrogenase, mitochondrial	54751	111	0.018	-1.29
83	DHE3_MOUSE	Glutamate dehydrogenase 1, mitochondrial	61640	80	0.021	-1.18
84	CAZA2_MOUSE	F-actin-capping protein subunit alpha-2	33118	490	0.029	1.08
85	A2ARF6_MOUSE	GTP:AMP phosphotransferase AK4, mitochondrial (Fragment)	12620	85	0.032	1.19
86	MDHC_MOUSE	Malate dehydrogenase, cytoplasmic	36659	461	0.045	1.12
87	ACON_MOUSE	Aconitate hydratase, mitochondrial	86151	52	0.049	-1.13
88	PSPC1_MOUSE	Paraspeckle component 1	58835	37	0.049	1.12

[a]Spot No assigned manually.
[b]Protein name identified by MALDI-TOF-MS/MS.
[c]Theoretical molecular weight of the protein (s).
[d]The ratio in spot intensity from 3xTg-AD mice treated with loganin compared with the control mice. N=6 for each group.

Supplementary Table 2. The usages of the primary antibodies.

Antibody	Type	Dilution	Application
6E10	mAb	1:400	IHC
APP	mAb	1:3000	WB
ADAM10	pAb	1:3000	WB
BACE1	mAb	1:1000	WB
IDE	pAb	1:3000	WB
$pTau^{S396}$	mAb	1:100	IHC
$pTau^{S262}$	pAb	1:50	IHC
PGAM1	mAb	1:3000	WB
ENO1	mAb	1:3000	WB
1433Z	pAb	1:3000	WB
SYN2	mAb	1:3000	WB
Cplx2	pAb	1:3000	WB
α-tublin	mAb	1:6000	WB
β-actin	mAb	1:3000	WB
